# Barriers Related to the Identification and Satisfaction of the Sexual Needs of Nursing Homes’ Residents: A Narrative Review

**DOI:** 10.3390/ijerph22081163

**Published:** 2025-07-22

**Authors:** Anna Castaldo, Jesus Francisco Javier Leon Garcia, Alessandra D’Amico, Giulio Perrotta, Stefano Eleuteri

**Affiliations:** 1 ASST Centro Specialistico Ortopedico Traumatologico G. Pini-CTO, 20122 Milano, Italy; anna.castaldo@unimi.it; 2 Fondazione IRCCS Ca’ Granda—Ospedale Maggiore Policlinico, 20122 Milano, Italy; jesusleon.rn@gmail.com (J.F.J.L.G.); kale2254@gmail.com (A.D.); 3 Department of Human and Social Sciences, Universitas Mercatorum, 00186 Rome, Italy; info@giulioperrotta.com

**Keywords:** older, sexual needs, nursing home, healthcare attitude, barriers, sexuality, intimacy

## Abstract

Background: Sexuality is a central aspect of being human, even if people experience it in different ways in various stages of life. Sexuality in older people may be expressed, as well as affection, companionship, touch, and physical contact. However, older peoples’ sexual needs are not properly considered by themselves, caregivers, or healthcare professionals. Reviews on barriers related to identification and satisfaction of sexual needs of people living in nursing home are scarce. In this scenario we intended to summarize the state of evidence regarding sexual need identification and satisfaction among older people living in nursing homes and possible barriers that could limit sexual need identification and satisfaction. Methods: We carried out a narrative review. The included studies responded to the research question, using the following key words: nursing homes, sexuality or sexual need, or sexual behavior, older people. Searched databases included PubMed, Embase, CINAHL, PsycInfo, and Scopus. Results: After searching and screening we included 22 studies, finding three main topics: 1. identification of sexual needs by residents and healthcare personnel attitude and practice; 2. barriers and reasons hindering the identification of sexual needs; and 3. manifestation and satisfaction of sexual needs. Conclusions: The findings showed that nursing homes’ residents have different sexual needs, but there are many organizational, educational, and cultural barriers and negative attitudes of healthcare personnel. Supporting nursing home residents to express their sexual needs is a challenge for the healthcare professionals and managers of nursing homes.

## 1. Introduction

According to the World Health Organization, “Sexuality is a central aspect of being human throughout life and compasses sex, gender identities and roles, sexual orientation, eroticism, pleasure, intimacy and reproduction. Sexuality is experienced and expressed in thoughts, fantasies, desires, beliefs, attitudes, values, behaviors, practices, roles and relationships. While sexuality can include all these dimensions, not all of them are always experienced or expressed. Sexuality is influenced by the interaction of biological, psychological, social, economic, political cultural, ethical, legal, historical, religious and spiritual factors” [1].

More than 30% of people over 70 years old remain sexually active [2]. Aging can change intimate relationships in terms of frequency and nature [3]. While the sexual goal in young people is mainly the physical coitus, sexuality in older people may take the form of fondness, companionship, touch, and the need to have the sensation to be attractive as masculine or feminine. In other words, anything may foreshadow the sentiment of “feeling loved” [4]. Caregivers should recognize the changing nature of sexuality because the role of non-carnal sexuality assumes an ever-increasing importance in old people [5]. Maintaining satisfying sexual activity over the time is positively associated with life satisfaction in older people.

Well-being in older people could be promoted by way of a person-centered approach, including relevant interventions for a fulfilling sexual life [6]

Most of older people prefer dwelling in their own home, and they are reluctant to live in a nursing home [7,8] However, they prefer to move into a residential facility when their care needs are increasing [9,10]. In nursing homes where person-centered care is implemented, residents report greater satisfaction and quality of life. This approach is based on the wishes and needs of the residents [11].

Sexual and affective needs continue to be fundamental for people who live in nursing homes, but it is also necessary to take into account the reason that pushed the person to live in a nursing home, as certain morbid conditions (such as neurodegeneration or brain injury) can significantly affect the libido and emotional and sentimental needs [12,13].

A review on the sexual expression of nursing home residents revealed that most care personnel have a good attitude regarding the sexual needs of residents; however, many others have a negative attitude, since they believe that sexuality assessment is not part of their role. Moreover, multiple factors make intimacy difficult, including resident and social factors, staff shortages, and lack of privacy [14,15]. Also, older people, especially those who live in nursing homes, may have misconceptions, prejudices, and taboos about aged sexuality and may believe that sex is only for young people [16].

Despite sexual expression remaining important for older people, it should be considered that the literature about people who living in nursing home is still rather poor and conflicting. This is due to several reasons, including stereotypes of ageism, residents’ vision, staff and knowledge attitude, ethical conflict, and prioritizing physical care needs [14,15,16].

Although studies in older sexuality are increasing over time, reviews that summarize possible barriers related to identification and satisfaction of sexual needs of people living in nursing homes are poor and out of date [15,16,17]. Therefore, it is relevant to know the ways of identifying the sexual and affective needs of nursing home residents and how they satisfy their own needs. Nonetheless, it is important know health personnel’s attitude and practices about assessment and management of the sexual needs of NH residents and possible barriers.

The aim of this review was to know and summarize the findings about NH residents’ sexual needs and barriers related to identification and satisfaction among older people living in nursing homes.

## 2. Materials and Methods

A narrative review was conducted from December 2022 to January 2023.

The research questions were, How and by whom are nursing home residents’ sexual needs considered and assessed? How do NH residents satisfy their sexual needs? What are the barriers and reasons to take into consideration?

Inclusion criteria for this review were as follows: articles in Italian, Spanish, German, and English languages and articles published within the past 10 years. Age was not defined; rather, articles whose target population was older people were considered.

Exclusion criteria included articles concerned with sexual abuse of residents and/or caregivers, as the topic focuses on sexual needs like the prevention of abuse and not the act itself. Articles whose target population was distinct from the older population and those focused on healthcare personnel’s sentiments were also excluded.

Databases inquired were PubMed, Embase, CINAHL, PsycInfo, and Scopus. We used the following keywords: nursing homes, sexuality or sexual need, or sexual behavior, older people. The research strategy is presented in Table 1.

Three reviewers (ADA, RG and JL) analyzed the full text of each included study and extracted the main data, including author, year, aim, type of study, participants, outcome, main findings, note.

Studies were grouped based on their design and the topic. Any discrepancy was discussed together and with another researcher (AC). Finally, a researcher expert in the topic (SE) assessed and reviewed the extraction findings. The overall review process lasted about 12 months. At the end of review synthesis, no other relevant studies had been identified.

## 3. Results

### 3.1. Study Selection and Study Characteristics

**Figure 1:**
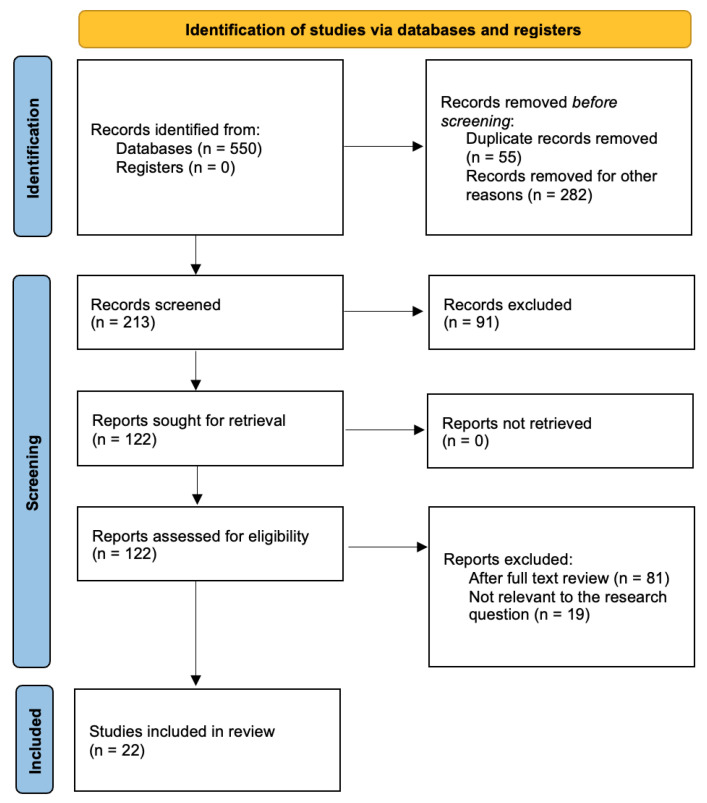
Flow diagram of review process.

The electronic searches yielded a total of 550 records. After applying filters and removing duplicates, a total of 213 records remained. We identified 41 records as potentially eligible, and we screened full texts for suitability to the research questions. Finally, 22 studies were included in the review. A flow chart of identification, screening, and selection of studies is illustrated in Figure 1 according on the PRISMA framework [18].

We identified three main topics: 1. identification of sexual needs by residents and healthcare staff attitude and practice; 2. barriers and reasons hindering the identification of sexual needs; and 3. manifestation and satisfaction of sexual needs.

The included studies were of qualitative (*n* = 11) or quantitative design (*n* = 11) and were conducted in the following geographical areas: America (*n* = 7), Europe (*n* = 10), Oceania (*n* = 4), Asia (*n* = 1). Details about the studies, including aim, design, participants, and main findings are reported in Table 2. Moreover, they are extensively explained in the online Supplementary Materials.

### 3.2. Identification of Sexual Needs by Residents and Healthcare Staff Attitude and Practice

Sexual needs in nursing home residents are often underrecognized due to a combination of personal inhibition and staff discomfort or lack of training. Residents often perceive sexual needs as belonging to earlier life stages and do not commonly express their needs openly. Moreover, healthcare personnel rarely initiate discussions about sexual needs; instead, they often focus on physical health concerns such as medication side effects, incontinence, or pain, which might impact sexual expression [19,20].

Residents’ attitudes reflect internalized ageism stereotypes or a sense that sexuality is irrelevant or inappropriate in later life, leading to underreporting [21,22]. Family members often have ambiguous expectations about sexual expression appropriateness in care settings, creating uncertainty for both staff and residents [23,24].

Healthcare staff attitudes and practice vary considerably. Assessments of sexual health in aged care facilities have been found to occur infrequently, and typically only in reaction to problematic behaviors rather than as part of holistic routine [20]. A systemic undervaluation of sexuality-related needs was evidenced by the fact that fewer than one-third of facilities reported gathering information on sexual orientation, sexual health, or sexual needs [20].

The implementation of structured assessment tools, such as the Sexuality Assessment Tool (SexAT), has been suggested to enhance care planning and promote consistent staff training and policy [19]. Nonetheless, adoption of such tools remains minimal, and most facilities lack designated personnel or systematic procedures to support the sexual expression of residents [25].

Institutional policies, whether present or absent, have a measurable impact on staff attitudes and practices. Rigid policy frameworks sometimes limit individualized care. Facilities without formal policies on sexuality were paradoxically associated with more open and positive staff attitudes, likely reflecting a less prescriptive and more resident-centered ethos [26].

Knowledge and education levels influence staff views about residents’ sexuality positively or negatively. Higher knowledge scores and long experience in nursing roles correlate with more supportive attitudes [27]. Cultural and religious backgrounds also play a relevant role; for example, some staff identifying as Islamic/Muslim report differing attitudes shaped by cultural norms [25].

One qualitative study found that understanding of older adults’ sexuality among nursing staff was generally limited, particularly among younger, less educated, and more religious individuals [24]. This study reports that only 13 out of 26 knowledge questions were answered correctly by more than half the participants; the findings reflect significant gaps in education and awareness [24].

In the case of LGBTQ+ residents, authentic and respectful recognition of identity, rather than performative or exaggerated gestures, was emphasized as fundamental to the experience of safe and affirming care. This highlights the importance of natural, sincere interactions over superficial inclusion efforts [23].

Healthcare professionals often experience discomfort discussing sexuality, sometimes perceiving sexual expression as inappropriate or threatening to professional boundaries [28]. This discomfort leads to evasion or dismissiveness, discouraging residents from expressing needs. Sexuality workshops could improve staff comfort and promote respectful dialogues [19,27]

Attention to diversity issues, including LGBTQ+ residents’ needs, remains limited but essential. Same-sex couples often face covert discrimination and lack recognition, negatively impacting their sexual well-being [19].

For instance, older lesbian and gay couples have been reported to anticipate covert discrimination in long-term care settings and often describe the need to expend emotional energy to safeguard their identities and relationships. This underscores the tension between the desire to be treated equally and the necessity of being acknowledged for unique identity and relational dynamics [23].

### 3.3. Barriers and Reasons Hindering the Identification of Sexual Needs

Structural, social, and attitudinal barriers obstruct free communication. Lack of privacy emerges as a predominant issue across multiple studies. Institutional configurations and shared environments lead facilities to prioritize safety and supervision over private spaces, restricting intimacy opportunities [29,30,31,32].

Residents feel reluctant or ashamed to express sexual needs, fearing judgment [33,34]. Families can act as significant gatekeepers, at times limiting residents’ autonomy, especially for individuals with cognitive impairments such as dementia. In such cases, ombudsmen often intervene to mediate conflicting views between families and facilities [30].

Staff avoid addressing sexual matters due to discomfort, lack of training, or institutional taboos [31,35]. Sex and intimacy remain taboo topics in aged care, and staff often lack the confidence to initiate conversations [34,36].

The invisibility of sexuality as a care topic is compounded by poor communication between care teams and residents, reinforcing its taboo nature [21,35]. Residents’ expressions of sexuality may range from denial or nostalgia to subtle affirmations of ongoing desire, reflecting a complex and diverse set of experiences [29].

Unspoken moral judgments by staff often shape the management of residents’ sexual behavior, particularly in the absence of formal ethical policies. Ad hoc decisions may result in inconsistent and sometimes exclusionary responses [37].

Many nursing homes lack sexuality-supportive policies and staff training, limiting sexual expression and satisfaction [25,30]. Moreover, family also act as gatekeepers, discouraging sexual expression [22,24].

Homophobia and heteronormativity suppress same-sex relationships and sexual expression, alienating LGBTQ+ residents [32,35]. Stigma and previous experiences of discrimination, especially toward non-heteronormative sexuality, are additional barriers faced by LGBTQ+ individuals, while physical frailty and cognitive decline are often cited as justifications for restricting sexual expression [23,33].

### 3.4. Manifestation and Satisfaction of Sexual Needs

Despite many barriers, nursing home residents find different ways to express and satisfy sexual and intimate needs. Younger residents (typically 58–67 years) are more likely to engage in intercourse; older residents express sexuality via non-penetrative forms such as masturbation, affectionate touch, or emotional intimacy [29,33,38]. Marital status influences sexual activity, with partnered residents reporting higher engagement [39].

Reports suggest that over half of surveyed residents experience sexual tension, and about one-fourth remain sexually active. Such expressions are more common among men and younger residents, although emotional intimacy, such as holding hands and sharing affection, is often prioritized over sexual intercourse [33].

Older women often have acknowledged ongoing desire, many of them describe sexuality as having receded in importance due to past familial roles and widowhood. Nonetheless, emotional connection and tenderness are still deeply valued [36].

Affectionate behaviors include holding hands, flirting, affectionate dialogue, and subtle seductive behaviors toward staff or residents [21,37]. Sexual needs extend beyond physical acts to include emotional and social dimensions such as companionship and tenderness.

Sexual activity is associated positively with overall quality of life and psychological well-being [28,40]. Nursing homes providing modifications in the care environment, including the creation private rooms or “dignified spaces” and visitor accommodations facilitate greater intimacy, helping residents meet sexual and affective needs more effectively [26,38,39]. An example is the use of a “Family Room” in a U.S. veterans’ facility, which enabled about 10% of residents to spend private time with partners in a more home-like setting [38].

Facilities that adopt tools like the SexAT solicit resident feedback on intimacy-related satisfaction and the impacts of medication [19].

Staff education programs have demonstrated measurable improvements in attitudes and understanding, contributing to better outcomes for residents [27]. It is reported that nursing aides showed increased knowledge and openness after attending sexuality workshops, correlating workshop participation with improved quality of life for residents [27].

Dementia care presents unique challenges. Spouses and staff often redefine intimacy emphasizing non-verbal affection as gestures of care and emotional presence rather than sexual intercourse [38]. Such expressions often include touching, cuddling, and frequent visits, underscoring the enduring importance of love and physical closeness [22]. Better staff education improves attitudes and person-centered care approaches for the sexual needs of people with dementia [19,22,34]. Summary characteristics of the studies included in the review is displayed in Table 2.

**Table 2:** Summary characteristics of the studies included in the review.

No.	Reference	Country	Aim	Design	Participants	Key Findings
1	[23]	Canada	Expectations of same-sex couples regarding LTC and home care	Qualitative (Grounded Theory)	12 couples (24 participants)	Concerns about covert discrimination; energy spent protecting identity; need for recognition and respect from caregivers
2	[38]	USA	Implementing private guestroom for intimacy	Case study	CLC residents	Policy and care barriers overcome to provide intimacy space; anecdotal evidence suggests positive outcomes
3	[24]	Belgium	Nursing staff’s knowledge and attitudes	Cross-sectional survey	1166 nursing staff	Low knowledge; conservative attitudes linked to low education, age, religiosity
4	[20]	Australia	Assessment of residents’ sexual health/needs	Quantitative (survey)	1094 nurse managers	Assessments rare, focused on disruptive behavior; for-profit homes more proactive
5	[33]	Poland	Psychosexual needs in nursing homes	Face-to-face survey	85 residents	Emotional needs prioritized; sexual activity low; desire still present
6	[25]	Belgium	Sexual rights support in aged care	Quantitative (survey)	69 facilities	Very few had policies; poor staff training; limited support for sexual expression
7	[28]	Belgium	Prevalence of sexual activity in elderly	Observational (Prevalence study)	511 participants (45 in NH/ALF)	NHs are barriers; sexually activity linked to better quality of life
8	[39]	France	Hidden sexual behaviors	Quantitative (survey)	300 nursing students	Common behaviors: affection, seductive actions, taboo perceptions
9	[37]	New Zealand	Ethical issues in sexuality management	Qualitative study	4 individuals	Absence of ethical frameworks; privacy symbolic, not real
10	[19]	Australia	Develop sexuality assessment tool (SexAT)	Mixed methods	Staff, residents, families	Tool includes policies, training, environmental factors
11	[40]	USA	Barriers to intimacy in assisted living	Qualitative	23 people	Barriers include norms, privacy; residents use subtle behaviors
12	[21]	Canada	Family/resident views on sexual expression	Qualitative	Residents + families	Lack of clarity on norms; need for privacy and communication
13	[30]	USA	Ombudsmen perspectives on sexual expression	Qualitative	31 ombudsmen	Ombudsmen support staff/residents; need for education
14	[34]	USA	Policies and staff reactions to sexuality	Observational	91 homes	Staff discomfort common; sexuality often seen as problematic
15	[31]	Spain	Resident and staff views on barriers	Qualitative	53 staff, 47 residents	Top barrier: lack of privacy; also, taboo, illness, silence
16	[36]	Spain	Older women’s lived experience of sexuality	Qualitative	20 women	Sexuality shaped by past roles; limited post-widowhood expression
17	[22]	Netherlands	Spouses’ experience of intimacy with partners in dementia care	Qualitative	9 spouses	Intimacy redefined; barriers include space, privacy
18	[26]	Netherlands	Staff attitudes and organizational influence	Observational	191 care staff	Higher education and knowledge = better attitudes; policy impact mixed
19	[35]	Australia	Rights and discourses on sexuality in aged care	Qualitative	42 participants	Staff call for normalization; community emphasizes sexual rights
20	[29]	UK	Addressing sexuality among older residents	Qualitative	16 participants in 2 homes	Expressions vary: denial, nostalgia, openness
21	[32]	Brazil	Nursing team’s response to elderly women’s sexuality	Qualitative	18 staff	Privacy issues, institutional control, homophobia limit expression
22	[27]	Taiwan	Effect of sexuality workshops on staff/residents	Quasi-experimental	68 aides, 100 residents	Workshops improved knowledge, attitudes, and resident well-being

## 4. Discussion

The overall objective of this review was to examine the literature on sexuality perceptions and satisfaction in nursing home residents.

The selected literature was analyzed based on three different thematic areas (identification of sexual needs, barriers, and manifestations of subjective satisfaction), from which emerges the lack of a structured program based on psychoeducational and clinical interventions. Clear critical issues also emerge regarding the ability of care staff to manage the emotional/affective and sexual issues of users and the absence of a precise directive regarding the use of operational and organizational tools.

In Table 3 are reported the main strengths and weaknesses of each selected study, related to three thematic areas.

Sexual needs of nursing home residents are diverse and enduring. A comprehensive response requires the integration of appropriate policies, ongoing staff training, and intentional environmental design to foster both dignity and expression [41].

Considering the findings, we can affirm that a global approach towards nursing home residents is lacking, and there are some aspects neglected by most healthcare personnel, probably due to stereotypes and inexperience in determining subjective needs and their best functional management [42].

In addition, we assume that continuous education towards sexuality of people living in long-term facilities is still missing. For many healthcare professionals, the sex and sexuality of patients, especially if they are older, are issues that they may be reluctant to address or to engage [43]. Stigma associated with elderly sexuality probably makes it still more difficult to take care of this aspect in this specific setting [42], especially if the topic of sexuality focuses on non-heterosexual orientations [44,45,46].

We consider that healthcare personnel training to identify and to fulfil specific sexual needs could be useful to overtake stereotypes and improve the quality of life of older people in line with other research, which considers sexuality one of the primary needs, together with activities and free time, autonomy and independence, interpersonal relationships, and religious faith and spirituality [11].

Even if training is not costless for an organization, we consider that it is necessary to have healthcare professionals capable of assessing residents’ needs in all aspects. Therefore, they could implement their interpretative skills to best define the strategies promoting the satisfaction of such needs but also the limits and boundaries necessary to guarantee well-being [17,47] and to reduce the ever-increasing rate of abuse and violence [48]. Indeed, we could assume that fulfillment of sexual need could reduce episodes of psychomotor agitation. As we stated in Section 2, it is important to understand how sexual education can increase the prevention of abuse, intended as a sexual health indicator, even if, in our review, we felt it was not useful to concentrate on the abuse itself.

Given increasing life expectancy and quality of life, it is reasonable to expect that people will be sexually active longer than before.

It is therefore essential to work towards promoting better knowledge and sharing of these affective, sentimental, and sexual aspects through specific education [43,49,50] with or without the use of technologies [51,52,53].

All healthcare professionals require education, aligned to their own discipline, to promote sexual satisfaction among older people [43,53].

An issue that should be considered is the lack of physical intimacy, which a couple needs to express tenderness or engage in intercourse; in our opinion, this intimacy could be created by either building new spaces or changing working organization, especially in this specific context, in which nurses do not routinely face life-threatening conditions.

This review has some limitations. Selected keywords are limited, so the review focused just on specific aspects of sexuality. The studies included in this review were mainly based on qualitative design, and they were published until 2023, as no other studies were found at the end of review process, which lasted about one year.

Moreover, most of the studies were conducted in Western countries; therefore, the generalizability of the results, especially to Asian or African countries, may be limited.

## 5. Conclusions

Sexual and reproductive rights are an essential pillar of overall health and well-being [53].

From this review it clearly emerged that nursing homes’ residents have different sexual needs and are oriented both towards sexual and affective/sentimental themes. Most of the obstacles are due to organizational and educational issues and to negative attitudes of healthcare personnel. Moreover, the absence of validated, structured intervention programs increases the subjective interpretation by individual operators and therapists, so we hope that our review can help in identifying intervention guidelines, both in terms of recognition of needs and of intervention through specific operational tools.

In the future perspective, it is necessary to commit to working on these current critical issues that favor the existence of dysfunctional factors and barriers that prevent the enjoyment of these subjective rights, so that sexual rights can also be enjoyed and guaranteed to this vulnerable and fragile population.

## Figures and Tables

**Table 1:** Research strategy.

	Research Strategy
PubMed	(“Sexuality”[Mesh] OR “Sexual Behavior”[Mesh]) AND (“Nursing Homes”[Mesh] OR “Residential Facilities”[Mesh])
Embase	‘nursing homes’ AND ‘sexuality’/exp AND (‘sexual satisfaction’/exp OR ‘sexual behavior’/exp)
CINAHL	TX nursing home AND TX sexuality AND TX sexual behavior
Psycinfo	(nursing homes and sexuality and sexual behavior).af.

**Table 3:** Strengths and weaknesses of the studies included in the review, in relation to the thematic areas. Thematic area: 1. Identification of sexual needs (ISN): Yes or No. 2. Identification of barriers that prevent the identification of sexual needs (IB): Yes or No. 3. Expression of satisfaction by users (ES): Yes or No. 4. Strengths (1a: positive evaluation of needs, 2a: detailed evaluation of the emotional profile, 3a: operational evaluation of tools and barriers). 5. Weaknesses (1b: underestimated or absent negative evaluation of needs, 2b: superficial or absent evaluation of the emotional profile, 3b: poor or absent evaluation of tools and barriers).

Reference	ISN	IB	ES	Strengths	Weaknesses
[23]	Yes	Yes	No	1a	2b, 3b
[38]	Yes	Yes	No	2a, 3a	1b
[24]	No	No	Yes	1a	2b, 2c
[20]	No	No	Yes	1a, 3a	2b
[33]	Yes	Yes	No	1a, 2a	3b
[25]	No	No	Yes	1a, 3a	2b
[28]	Yes	Yes	No	1a, 3a	2b
[39]	Yes	Yes	No	1a, 2a	3b
[37]	No	No	Yes	1a	2b, 3b
[19]	Yes	Yes	No	3a	1b, 2b
[40]	Yes	Yes	No	1a, 3a	2b
[21]	No	No	Yes	1a	2b, 2c
[30]	Yes	Yes	No	1a, 3a	2b
[34]	No	No	Yes	1a	2b, 3b
[31]	Yes	Yes	No	3a	1b, 2b
[36]	Yes	Yes	No	1a	2b, 3b
[22]	Yes	Yes	No	1a	2b, 3b
[26]	No	No	Yes	1a, 3a	2b
[35]	Yes	Yes	No	1a	2b, 3b
[29]	No	No	Yes	2a	1b, 3b
[32]	Yes	No	Yes	3a	1b, 2b
[27]	Yes	No	Yes	1a, 1b	2c

## Data Availability

Data available upon reasonable request.

## Supplementary Materials

The following supporting information can be downloaded at: https://www.mdpi.com/article/10.3390/ijerph22081163/s1, Table S1: Details about the studies, including aim, design, participants, and main findings.
