# Supplementary material for: Barriers Related to the Identification and Satisfaction of the Sexual Needs of Nursing Homes’ Residents: A Narrative Review
Source: Int J Environ Res Public Health. 2025 Jul 22;22(8):1163. doi: 10.3390/ijerph22081163 (PMC12386229; doi:10.3390/ijerph22081163)
Supplement: Supplementary file 1 [file ijerph-22-01163-s001.zip › ijerph-3703814-supplementary.pdf]

Table S1. Details about the studies, including aim, design, participants, and main findings.

| | |
|---|---|
| Title | "Could We Hold Hands?" Older Lesbian and Gay Couples' Perceptions of Long-Term Care Homes and Home Care |
| Author (Year) | Charles Furlotte, James W. Gladstone, Robert F.Cosby and Kerri-Ann Fitzgerald (2016) |
| Aim | what do couples think about entering a long-term care (LTC) home and using home care services? What are their expectations? What kind of care do they feel they need, may come to require, and deserve |
| Met_Study Design | The research team utilized a grounded theory approach guided by the interpretive paradigm (Strauss & Corbin, 1998 ) where thematic categories were derived inductively from the data. The team sought same-sex couples (female and male) to participate in an interview, either in person or by telephone (approximately 1–1 1/2 hours in length per participant).<br>The research team identified themes through dialogue with research participants and were not necessarily restricted to responses given to any one question.<br>The team also utilized snowball sampling and encouraged participants to refer appropriate others to the study.<br>Researchers recruited participants through posted details about the research through LGBTQ2-friendly identified websites (Gay Okanagan,McMaster University). The team also utilized snowball sampling and encouraged participants to refer appropriate others to the study (Patton, 1990 ). The research coordinator made initial contact with one member of the couple who contacted the coordinator by telephone and/or email. The research coordinator then explained the nature of the study in detail, and if the participant was still interested in participating, an interview was arranged. Couples were presented with the questions from the interview guide prior to the interview so they could discuss in advance the issues of home care and LTC homes together, to think about their responses beforehand, and know what they would be asked. The coordinator gave the participants the option of being interviewed individually or in dyads. Of the 24 participants, 22 opted to be interviewed in coupled dyads, and two opted to be interviewed individually (due to scheduling conflicts between the interviewers' and participants' schedules). Due to geographic distance between interviewers and participants, the researchers conducted nine interviews over the telephone using digital technology to record the interviews, with both participants on the line at the same time. Seven of these nine telephone interviews were conducted with dyads, and two were conducted with partners of the same couple separately. The research team conducted four face-to-face interviews with local couples with both parties of the relationship present at the same time.<br>Three of these interviews were conducted in the participants' homes and one interview in a space requested by the couple.<br>The team offered participants an honorarium of $25 to participate.<br>The team gathered qualitative data using an interview guide that included questions about their health, family, employment, experiences of discrimination, health services, LTC homes and home care, specific needs they might have, and suggestions for improving care.<br>Social workers previously trained in interviewing skills conducted the interviews. |
| Met_Population | At least one person in each coupled relationship was age 50 years or older. There were no other inclusion criteria. The research team drew findings from qualitative data gathered from 12 couples (24 individuals making up four male same-sex couples and eight female same-sex couples) living in Ontario, British Columbia, and Alberta, Canada. One partner in one of the couples identified as transgender. The participants ranged in age between 39 and 75 (M = 63.58 years, median age 65) |
| Met_Intervention | Researchers recruited participants through posted details about the research through LGBTQ2-friendly identified websites (Gay Okanagan, McMaster University). The team also utilized snowball sampling.<br>The team gathered qualitative data using an interview guide that included questions about their health, family, |

| | |
|---|---|
| | employment, experiences of discrimination, health services, LTC homes and home care, specific needs they might have, and suggestions for improving care Social workers previously trained in interviewing skills conducted the interviews. |
| Met_Primary_outcome | expectations, concerns, and needs regarding long-term care (LTC) homes and home care services of 12 older lesbian and gay couples living in Canada. |
| Results | qualitative findings are organized around four major categories: discrimination; identity; expenditure of energy; and nuanced care. When thinking about the prospect of accessing home care services or an LTC home, participants expressed concerns about covert discrimination against lesbians and gays; loss of social buffers as they age; and diminished ability to advocate for oneself and one's partner. In terms of identity, participants anticipated risk over disclosing one's sexual identity and noted the central importance of being identified as a partner in a coupled relationship; they also acknowledged the importance of having access to reference groups consisting of other gay seniors.<br>Participants were wary of discrimination that they might face if their or their partner's health declined and they required home care services or entered an LTC home.<br>Three properties were associated with this category: covert discrimination; loss of buffer; and diminished ability to advocate.<br>Discrimination: most of the participants did not expect to encounter overt discrimination, if only because legislation over the years has protected them from being targeted.<br>Their concern, rather, was that they would be subjected to a more covert type of discrimination if they entered an LTC home or if they used home care services. As Natalie put it, "you can't legislate morality." This type of discrimination could come about in an indirect way, such as being disparaged, even if the participant wasn't physically present.<br>A few participants saw their professional status at work as being a potential buffer that would protect them against discrimination. Their concern was that as they aged and if their health declined to the point that they needed home care, this buffer would be removed and they would be more open to discrimination.<br>Several participants had advocated for themselves or for others over the years. In some cases, participants worried that declining health would cause them to lose their ability to advocate for themselves or their partners.<br>Some participants felt that discrimination would be even more likely to be experienced in smaller or rural communities. This could be related to greater acceptance typical of, or to the greater likelihood of social support being available in, a more cosmopolitan centre.<br>Identity: most participants were clear about their need to retain their identity as a gay or lesbian person.<br><br>Expenditure of Energy<br>Participants reported having to expend a great deal of energy to ensure their well-being in care environments.<br>Three properties were associated with this category: hiding identity; assessing their environments for discrimination, and placating or educating others. Participants actively looked out for cues signalling emotionally uncomfortable situations for which they tried to be prepared. This was expected to continue if home care and LTC home services were utilized.<br>Unlike other themes that emerged from interviews where participants spoke about their fears concerning |

| | |
|---|---|
| | discrimination or their level of comfort with identifying themselves as lesbian or gay, this theme refers to energies that were expended by participants in their efforts to protect themselves or their partners from actual or perceived threats.<br>Nuanced Care<br>Three properties were associated with this theme: feeling comfortable with health care providers; having providers feeling comfortable with participants; and duality of need. It was important for the study partici-pants to feel comfortable with their health care providers. This need for comfort went beyond having a health care provider who didn't convey prejudice. Participants were looking for health care providers who could respect their lifestyle and empathize with their concerns around receiving care from someone who was not lesbian or gay. At the same time, the desire was for the health care provider to act in a natural and sincere way and not to show acceptance in an overly solicitous or exaggerated manner. Pina, for example, referred to earlier years at work where she felt that her colleagues "wanted to be liberal but didn't get it ... they wanted to be kind but they didn't understand."<br>Her concern was that this history might repeat itself if she or her partner accessed home care or entered an LTC home.<br>For many participants, there was a duality between wanting to be treated just like any other person receiving health care services, and at the same time, wanting to be recognized as a lesbian or gay person with needs that were unique. |
| Note | These data provide some clues about how intersecting identities may lead to some of participants' perceptions of care. For instance, the confluence of sexual identity, gender, and race, along with ageism, are all important considerations that inform constructions of vulnerability associated with the sick role (Parsons, 1951 ); and these intersections may help account for the different care lesbian and gay older adults might experience.<br>Many participants were asking for services that would be no different from those afforded to heterosexual couples.<br>Recognition and acknowledgement of same-sex relationships is a first step to challenging current heterosexist assumptions in LTC homes and affirming older gay and lesbian people in care.<br>As articulated by some participants, non-heterosexist banter is welcomed, but this may require delicate attention. Participants also expressed a duality of need: lesbian and gay couples want to be treated just like any other person but at the same time, they want to be recognized as unique. |
| Title | Creating Space for Relationships |
| Author (Year) | Susan L. Maataoui and Jodi S. Hardwick Tessa S. Lundquist (2017) |
| Aim | this case study describes the rationale and process used to establish a private guestroom |
| Met_Study Design | case study |
| Met_Population | Healthcare System Community Living Center (CLC) residents |
| Met_Intervention | implementing a space for intimacy |
| Met_Primary_outcome | percentage of resident that used the space for intimacy |
| Results | Questions about resident safety, infection control, medication administration, nursing supervision, and furniture selection required review and guidance. Nursing Coverage for the veteran staying in the guestroom was one particularly difficult hurdle. Nursing management questioned how to balance privacy and care while a resident and partner were staying in the Family Room. Resolving this issue required extensive coordination with nurse managers and floor nursing staff. |

| | |
|---|---|
| | significant amount of time passed as the Family Room request made its way through committees, approvals, and policy discussions. During this period VA Maine instituted other artifacts of culture change (Bowman, 2006) to address dignity and self-determination, such as encouraging staff to wear street clothing, training staff in dementia-capable care, and establishing care practices that respected individual preferences. The use of aromatherapy, instituting summer barbecues and weekly breakfast buffets, and choosing "neighborhood names" were all part of an ongoing effort to create a more homelike experience for the veterans in residence.<br>During this waiting period, staff observed many missed opportunities for veterans to spend quality time with their loved ones, away from the milieu and peers. For instance, on one occasion a spouse expressed a desire to spend private time with her husband.<br>The highly decorated World War II veteran had severe PTSD from the trauma he experienced during battles on the beaches of Normandy. In the past, holding and snuggling him helped calm his fears and reminded him he was safe.<br>The Family Room has a double bed, dresser, writing desk, TV,and a small dining set, and a private bathroom stocked with fluffy towels and luxury bath products. Beautiful pictures decorate the walls and a large window lets in morning light. It is more akin toa hotel room than a hospital room, even with a few additions for resident safety such as a nurse call bell. The unit social workermanages reservations, and the room is available for either one or two night stays. Meals are delivered to the room if requested, and individual arrangements are made for medication and nursing care.<br>Since February 2016, approximately 10% of Patriots Place residents and their partners have used the Family Room to spend time together. Partners have also stayed in the room to be close to a loved one in hospice care. Although formal assessment to evaluate the benefits of having the Family Room has yet to be done, anecdotal evidence from staff and families suggest that it was worth the wait.<br>Helping staff become comfortable with the possibility that residents may engage in intercourse in the Family Room is a work in progress. VA Maine continues to discuss how the room is used, and to consider the intimacy needs of other CLC residents who may not have long-term partners or who do not reside on Patriots Place. |
| Note | Legally, there is no difference between genders in terms of the importance of capacity, though staff attitudes and adherence to policy may differ based on gender. Nursing home staff often conceptualize older women as more vulnerable and having less capacity to consent to intimacy (Ward et al., 2005). These attitudes and perceptions will be especially important to consider when designing family rooms in non-VA nursing homes, which likely have a varying distribution of male and female residents. |

| | |
|---|---|
| Title | Nurses' knowledge and attitudes toward aged sexuality in Flemish nursing homes |
| Author (Year) | Lieslot Mahieu, Bernadette Dierckx de Casterle, Jolien Acke,Hanne Vandermarliere, Kim Van Elssen, Steffen Fieuws and Chris Gastmans (2016) |
| Aim | to investigate nursing staff's knowledge and attitudes toward aged sexuality |
| Met_Study Design | Descriptive cross-sectional survey study |
| Met_Population | participants had to be working as nursing staff (head nurse, registered nurse,or nursing assistant) at one of the participating nursing homes<br>Nursing homes were assigned to one of nine mutually exclusive categories based on both their size (small,60 beds; medium, 61–120 beds; large,121 beds) and governance |

| | |
|---|---|
| | structure(public, private nonprofit, private profit). Out of each stratum, a proportionate random sample was taken, leading to a final selection of 202 nursing homes |
| Met_Intervention | Of the 2228 distributed questionnaires, 1358 were returned (return rate=61.0%). A total of 1166 questionnaires were included for analysis after exclusion of blank forms (n=166), forms that did not meet the<br>inclusion criteria (n=20), and forms that had no information completed on both the knowledge and atti-tudes components (n=6).<br>Analyses of attitudes were conducted for only 1149 questionnaires,<br>because 17 forms had attitude sections that were left completely blank. With more than 1000 individuals participating in this study, the number of participants was high enough to detect any clinically relevant association. As such, no formal power analysis was conducted.<br>In total, 43 nursing homes agreed to participate (overall participation rate=21.3%). The majority of the participating nursing homes were private nonprofit organizations (n=25; 58.1%). About 45%were medium sized (61–120 beds). All but three were officially recognized by the state for providing high-level residential care. All eligible nursing staff within these 43 nursing homes (n=2228) received an invitation letter, an information brochure, and a questionnaire nursing staff respondents who were older seemed to be more knowledgeable about older people's sexuality |
| Met_Primary_outcome | knowledge about older people's sexuality (measured with 26 questions using a true/false answer).Total scores could range from 26 to 78, with 26 being a perfect score. |
| Results | The overall participation rate was 52.3%.<br>The nursing staff sample was primarily female (92.5%). Participants' ages ranged from 18 to 65 years (mean=37.9 years, SD=11.0 years).<br>A large majority had at least 5 years of general work experience or work experience in caring for older people (77.8%and 73.8%, respectively). Approximately 90%of the respondents had not taken any continuing education courses addressing older people's sexuality during the past 3 years.<br>respondents had a mean knowledge score of 47.6 (SD=11.2), with a<br>range of 26 to 78;Only 13 out of 26 knowledge questions were answered correctly by more than half of the participants<br>ith regard to knowledge acquired, only three<br>reliable independent sociodemographic predictors were found, two of which also were significant in the<br>univariable analyses. These were age (p < 0.001) and number of continuing education courses in caring for older people (p=0.006). The third significant predictor of acquired knowledge related to the respondents' relational status (p=0.023). Respondents who were currently involved in a relationship appeared to be less knowledgeable about older people's sexuality than those who were not.<br>The nursing home's geographic location also seemed to have an effect on the level of knowledge acquired (p < 0.001).<br>More conservative attitudes were strongly associated with a lower level work position (p < 0.001), lower educational level attained (p < 0.001), higher religiosity self-rating (p < 0.001), self-identified Islamic/Muslim affiliation (p < 0.001), younger age (p < 0.001), and fewer years of work experience in general (p <0.001) or in caring for older people (p < 0.001). |
| Note | almost half of the nursing homes (n=48) stated that policy plans, re organizations, or management issues hindered their participation. Another significant barrier for participation was related to employee actors, such as shortage of staff and motivational problems (n=13).<br>Nursing staff's knowledge about older people's sexuality is rather limited |

| | |
|---|---|
| Title | Assessment of sexual health and sexual needs in residential aged care |
| Author (Year) | Linda McAuliffe, Michael Bauer, Deirdre Fetherstonhaugh and Carol Chenco (2014) |
| Aim | To investigate if, when and how assessment regarding residents' sexual health and needs occur within Australian residential aged care facilities. |
| Met_Study Design | The project was a postal survey of all 2766 Australian residential aged care facilities. A survey was developed specifically for the project and consisted of 21 items, with questions mainly requiring forced-choice tick-box responses. Questions asked whether the facility gathered information about residents' sexual health, sexual needs, intimacy needs, sexual orientation, disruptive sexual behaviour and/or sexual history and, if so, when the assessment occurred, who conducted the assessment, and how the information was gathered (by using a structured assessment form or by verbal means only). |
| Met_Population | Directors of nursing and nurse unit managers were targeted (rather than centre directors or administrators) due to their experience and knowledge. |
| Met_Intervention | A survey was developed specifically for the project and consisted of 21 items, with questions mainly requiring forced-choice tick-box responses. Questions asked whether the facility gathered information about residents' sexual health, sexual needs, intimacy needs, sexual orientation, disruptive sexual behaviour and/or sexual history and, if so, when the assessment occurred, who conducted the assessment, and how the information was gathered.<br>Participants were also asked to return a de-identified copy of the admission assessment form or sexual health/needs assessment form used by their facility so the form could be analysed for content relating to sexual health and needs. |
| Met_Primary_outcome | assessment of residents' sexual health and sexual need |
| Results | A total of 1094 completed surveys were returned, representing a 39.7% response rate.<br>Most respondents were female (90.7%), aged over 30 years old (98.1%) and held the position of director of nursing in their facility (58.5%).<br>By far the type of sexual information most often gathered concerned disruptive sexual behaviour (66.1%)<br>Less than a third of facilities reported gathering information about a resident's intimacy needs (31.7%), sexual health (24.7%), sexual needs (20.2%), sexual orientation (20.5%) or sexual history (13.7%).<br>Assessments most frequently occurred when a disruptive behaviour occurred (96.9%). However, facilities reported that assessments also commonly occurred when a family initiated discussion (89.7%), when a resident initiated discussion (87.3%), on admission (86.7%), whenrequested by a general practitioner (82.7%) or when the resident care plan was reviewed (80.5%). The most commonly cited 'other' occasions when assessments occurred were 'as required'/'when necessary' and 'when resident becomes sexually inappropriate'.<br>Assessments of sexual health/needs/preferences were most often performed by registered nurses (62.2%), followed by general practitioners/geriatricians (18.6%), enrolled nurses |

| | |
|---|---|
| | (16.4%), lifestyle/diversional therapists (16.4%) and personal care attendants/assistants in nursing (8.5%)<br>Only 34 respondents (3.1%) returned a de-identified copy of their facility's assessment form (one respondent returned two different forms).<br>Post hoc analyses revealed that for-profit facilities were more likely than not-for-profit facilities to gather information about residents' sexual needs (26% vs 18%; $\chi 2=8.4, P<0.01$),intimacy needs (38% vs 30%;$\chi 2=6.9, P<0.01$), sexual orientation (26% vs 19%;$\chi 2=5.2, P<0.05$) and disruptive sexual behaviour (73% vs 64%;$\chi 2=7.0, P<0.01$). For-profit facilities were also more likely than not-for-profit facilities to gather such information during the admission assessment(93% vs 84%;$\chi 2=8.0, P<0.01$) and during reviews of residents' care plans (88% vs 78%;$\chi 2=7.5, P<0.01$).<br>Larger facilities were more likely to gather information about residents' sexual orientation (25% for facilities with≥91beds vs 16% for those with 61–90 beds;$\chi 2=9.0, P<0.05$)and were more likely to gather information about residents' sexuality during the admission assessment (≥91 beds, 92%;<90 beds, 87%;$\chi 2=7.8, P<0.05$) and when the resident care plan was reviewed (≥91 beds, 89%; 31–60 beds, 76%; $\chi 2=13.7, P<0.05$).<br>Facilities with a higher proportion of residents with dementia (>75%) were more likely than those with fewer residents with dementia (26–50%) to gather information about residents' sexuality during the admission assessment (99% vs 83%;$\chi 2=15.3, P<0.05$) and when the care plan was reviewed (90% vs 75%;$\chi 2=8.0, P<0.05$).<br>In response to a question regarding survey format, over a third (39.9%) of respondents indicated they would have preferred an online survey. |
| Note | |

| | |
|---|---|
| Title | Psychosexual needs and sexual behaviors of nursing care home residents |
| Author (Year) | Bozena Mroczek,Donata Kurpas,Małgorzata Gronowska,Artur Kotwas,Beata Karakiewicz(2013) |
| Aim | The purpose of this study was to analyze psychosexual needs of nursing care home residents in Poland.<br>The authors attempted to answer the question 'how do residents satisfy their psychosexual needs?' |
| Met_Study Design | This study based on the face-to-face survey method, was performed by a properly trained pollster.<br>All interviews were conducted in the nursing care home, either in the residents' self-contained rooms or in a day room, with respect for the residents right to privacy and intimacy. |

| | |
|---|---|
| | The respondents answered all questions including those asking directly about intimate contacts; they did not avoid questions about masturbation. |
| Met_Population | The study was conducted in December 2010 and January 2011 in a nursing care home occupied by 223 residents.<br>The study inclusion criteria were: health state and self-reliance measured with the Barthel Index (BI).<br>The residents with a diagnosis of dementia, with the BI score below 40, and those who did not give their written consent for taking part in the study were excluded.<br>The authors excluded from the study 45 subjects in a critical condition (BI 0–20 points) and 72 individuals whose health state was described as moderate to severe (BI 21–40 points).<br>Additional exclusion criterion was a diagnosis of senile dementia.<br>Some 21 resident did not consent to take part in the study for their religious beliefs and reluctance to talk about human sexuality.<br>Some 85 residents of a nursing care home in Poland were interviewed.<br>The mean age was 72.2+-9.44 (58–92 year).<br>The women were older than the men (the mean age for women was 76.03+- 9.38,and the mean age for men was 73.71+-9.61) |
| Met_Intervention | This study, based on the face-to-face survey method was performed by a properly trained pollster.<br>All interviews were conducted in the nursing care home, either in the residents' self-contained rooms or in a day room.<br>The respondents answered all questions including those asking directly about intimate contacts; they did not avoid questions about masturbation.<br>The questionnaire concerned five socio-demographic variables: age, gender, marital status, education, and health problems.<br>It also demonstrated how these factors influence psychosexual needs andways to satisfy them.<br>the questionnaire concerned the importance of psycho-sexual needs, the feeling of sexual tension, deriving pleasure from sexual contacts, ways to relieve sexual tension, the possibility of satisfying psychosexual needs in nursing care homes, and social stereotypes about sexuality of the elderly.<br>The 5-point Likert scale was used to determine the importance of psychosexual needs, from the least (1) to the most important(5).<br>The 'needs' category included those essential for psychosexual<br>functioning |
| Results | The majority of the respondents(77.6%,66)choose the need for conversation as the most important (5points),then the need for mutual respect(75.3%,64),the need for tenderness(64.7%,55),and theneed for<br>getting support in any situation(61.2%,52).<br>The Need For Physical Closeness With Another Person(45.9%,39)and<br>the need for living together(33%,28)were regarded as the least important<br>(1point each) by most respondents.<br>Psychosexual needs were divided into two groups:mental(mental closeness,tenderness,conversation,respect,and support)and physical(physical closeness,intimate contacts,living together).<br>A moderate positive correlation was observed between the assessments of the needs' importance,higher importance of physical needs was accompanied by higher importance of mental needs(rxy=0.509,p<0.001). |

| | |
|---|---|
| | The Respondents With Motor Organ diseases and chronic disorders statistically significantly more often mentioned the need for: conversation,respect,tenderness,and mental closeness(p=0.0001)as the most important.<br>The need for living together was regarded as important significantly more often by the residents with motor organ diseases.<br>Regardless of the type of a disease, the needs for: physical closeness (mean 2.07, Me 1), intimate contacts (mean 2.53, Me 2) and living together (mean 2.27, Me 1) were perceived as not important (the lowest mean scores).<br>The analysis of the need hierarchy proved that gender had an effect<br>on the need for tenderness (p=0.012)–<br>all the men regarded it as important26%(9)or very important<br>73.5%(25).<br>Women,on the other hand, attached greater significance to respect (p=0.001) and support in any situation(p=0.016).<br>The analysis of other needs didn't prove statistically significant differences<br>between the women and the men.<br>There were no statistically significant correlations between such variables<br>as age, education or marital status, and ranking psychosexual needs at the<br>top of the need hierarchy (important and very important).<br>Sex was associated with sexual intercourse by 34% (29) of the respondents with the prevalence of: younger individuals (58–74year olds vs. 75–92 year olds; a statistically significant difference p = 0.043), men (47%, 16 vs. women 31.4%, 13), subjects with chronic disorders (40.6%, 15), and those with motor organ diseases (36.7%, 11). Sex was associated with marriage by 20% (17) of the respondents, most of whom were older individuals (75–92 years), women (19.6%, 10 vs. men 20.6%, 7), and those with chronic diseases (28.56%, 14). Some 16% (14) connected sex with kissing – they were mainly younger people (58–74 years) and women (23.5%, 12 vs. men 5.9%, 2). For 13% (11) sex was just a memory from their youth – mostly older individuals (75–92 years). Fewer than every tenth respondent associated sex with cuddling (6%, 5), or fascination with another person (6%, 5). About 5% (4) of the respondents did not associate sex with anything pleasant. There were no statistically significant differences (p > 0.05). Nobody associated masturbation with sex.<br>Over a half of the analyzed seniors (51%, 43) claimed that they felt sexual tension. The group with sexual tension comprised 61% (21) of men and 49% (25) of women. From the group of 43 subjects who experienced sexual tension, 14% (6) felt such tension once a week, and 86% (37) – seldom. Sexual tension was more common among younger individuals, men, and subjects with motor organ and chronic diseases (statistically significant difference, p < 0.05). It was demonstrated that sexual tension was decreasing with age of the respondents. There was a positive moderate correlation (rxy = 0.597, p < 0.001) between the variables.<br>The respondents having long-term partners constituted 16.5% (14) of all respondents, and felt sexual tension once a week (14.3%, 2) or seldom (64.3%, 9). In the group of singles (83.5%, 71), sexual tension was experienced once a week by 5.6% (4), and fewer than that by 39.4% (28) (the difference was statistically significant, p = 0.029). About 7% (6) of the residents with health problems felt sexual tension once a week, 41.2% (35) fewer and 10% (3) (those with motor organ diseases) more than once a week. Differences were statistically significant, p = 0.001.<br>About 24.7% (21) of the respondents remained sexually active and derived pleasure from sex. Individuals who experienced sexual tension no more than once a week, usually had sexual intercourse with their long-term partners – 27.9% (12), one man feeling sexual tension once a week admitted to masturbation, and another one to watching erotic films. Other ways to relieve sexual tension included diverting attention to other activities |

| | |
|---|---|
| | (60.5%, 26) and walking (7%, 3). Pleasure from sexual intercourse was derived by 24.7% (21) of the respondents, mostly those with motor organ diseases (40%, 12), and over a half of those having life partners (57.14%, 8). From among singles, 18.31% (13) were sexually active and derived pleasure from sex. This group consisted of men only, which shows that men are sexually active even if they do not have partners. The residents of all ages were sexually active, but younger individuals predominated (58–74 year olds vs. 75–92 year olds) (the difference was statistically significant, p = 0.011).<br>Not all nursing care home residents could satisfy their psychosexual needs. Only 39% (33) of the respondents said that they had such possibility. Meetings (41.2%, 35), cultural events (30.6%, 26), personal freedom and respect for privacy (22.4%, 19) are elements which create favorable conditions for meeting new people, having conversations and making plans for the future. Only 6% (5) of the residents claimed that they had talked about their psychosexual needs with their caregivers. |
| Note | The presented study was a concern to a limited number of the nursing care home residents in Poland. The respondents were not asked about their sexual health problems, which made it difficult to interpret the results proving the residents' low sexual activity. |

| | |
|---|---|
| Title | Sex and Intimacy Policy in Residential Aged Care Facilities in Flanders, Belgium |
| Author (Year) | Els Messelis, Michael Bauer (2019) |
| Aim | The extent to which Flemish aged care facilities comply with best practice in supporting residents' expression of their sexuality and specifc needs and gaps in facility practices, processes and policies which impact on residents' expression of their sexuality. |
| Met_Study Design | Survey |
| Met_Population | 750 aged care facilities (69 completed) |
| Met_Intervention | Sexuality Assessment Tool |
| Met_Primary_outcome | To investigate how supportive Flemish residential aged care facilities are of the sexuality and intimacy of their Dutch residents |
| Results | Identifying sexuality needs:<br>• resident,<br>• family,<br>• formal caregiver<br>12% acknowledged the availability of promotional or marketing material that indicated support for residents' rights to sexual expression.<br>Fifty-one and 61% percent respectively provided opportunities for residents to discuss the side effects of medications on sexuality<br>Only 30% provided residents an opportunity to discuss how satisfied they were with the facility's support for sexuality and any medical or other factors that might impact on sexuality<br><br>Reasons why the need is not recognised<br>No facilities used an assessment tool to identify residents' needs for sexual expression<br>How the need is expressed<br>Disinhibition, obscene gesturing, unwanted contact, abuse, stalking and sexual harassment.<br>Other<br>Thirty percent of facilities indicated that they offered residents information about sexual aids, lubricants, condoms and audio-visual materials on request, but only 20% have a designated and trained staff member who is able to support residents with sexuality issues |
| Note | CINAHL<br>There is a clear need for facilities to implement measures that improve opportunities for privacy, intimacy and expression of sexuality by developing specific policies and implementing staff education and training with input from all stakeholders such as nurses, family members and residents. Equally essential is that the professional |

| | |
|---|---|
| | development needs of staff with regards to sexuality and intimacy are assessed to allow for the development of appropriate policies that effectively guide staff responses in useful |

Extraction 2 (Identifying sexuality needs: resident, family, formal caregiver); reasons why the need is not recognised; How the need is expressed

| | |
|---|---|
| Title | Sexual Activity and Physical Tenderness in Older Adults: Prevalence and Associated Characteristics From a Belgian Study |
| Author (Year) | Adina Cismaru-Inescu, Bastien Hahaut, Stephane Adam, Anne Nobels, Marie Beaulieu, Christophe Vandeviver, Ines Keygnaert and Laurent Nisen. (2022) |
| Aim | To assess the prevalence and predictors of sexual activity and physical tenderness in a sample of older adults |
| Met_Study Design | Prevalence study |
| Met_Population | Belgium. 511 older adults >70 years living in the community, assisted living facilities, or nursing homes. Which 45 were from NH/ALF. |
| Met_Intervention | Interview using data from (UN-MENAMAIS) |
| Met_Primary_outcome | Current sexual activity and physical tenderness in the previous 12 months, sociodemographic characteristics, chronological age, subjective age, number of sexual partners, sexual satisfaction, attitudes toward sexuality in later life, quality of life, and lifetime sexual victimisation |
| Results | Healthcare professionals continue to have negative attitudes on later life sexuality, and living in a nursing home can be a barrier to sexual expression<br>Sexual needs is expressed being in relationship. Sexually active. Physical tenderness.<br>Other<br>85% of sexually active and 77.5% of sexually inactive individuals indicated a (very) good quality of life. |
| Note | No further data regarding NH/ALF residents was present in this study |

| | |
|---|---|
| Title | A study of hidden sexuality in elderly people living in institutions |
| Author (Year) | T. Darnaud, S. Sirvain, V. Igier, M. Taiton (2013) |
| Aim | to define a group of gestures, attitudes and behaviour which clearly underlie a sexual type of preoccupation |
| Met_Study Design | Survey |
| Met_Population | France. 300 first year nursing students at the end of first intership on a hospital ward or nursing home |
| Results | The topic is a taboo<br>How the need is expressed<br>Seductive attitudes towards the carers<br>intimate dialogue while being washed<br>undressing the students themselves with their eyes<br>kissing another elderly<br>singing romantic song<br>use pet name (sweetheart, my beauty, ecc)<br>holding hands<br>affectionate gestures<br><br>Other<br>Disinhibition, undressing and proposing sexual acts is more frequently betweend patients with cognitive disorder. |
| Note | |

| | |
|---|---|
| Title | Ethics, intimacy and sexuality in aged care |
| Author (Year) | Catherine Cook, Vanessa Schouten, Mark Henrickson, Sandra McDonald |
| Aim | To explore the ethical positions that inform conceptualisations of and responses towards intimacy and sexuality in residential aged care |
| Met_Study Design | Qualitative study |
| Met_Population | New Zealand. 4 participants: 1 RN, 1 healthcare assistant, 1 female resident, 1 femal relative. |
| Met_Intervention | Semi-structured interview |
| Met_Primary_outcome | to advance theoretical and ethical knowledge on intimacy and sexuality in the context of residential care |
| Results | Identifying sexuality needs by family (monitor residents ' relationships, assessing safety in a relationship between residents with dementia and formal caregiver (ensure residents wellbeing and autonomy; understanding people's present -day need for affection)<br>Reasons why the need is not recognised<br>often tacit moral judgments<br>Decision without formal opportunities for reflection, consultation or reference to ethical frameworks<br>Residents were not consulted in decision -making processes.<br>institutional unwritten rules<br>Lack of education, guidelines or team processes<br>Distress and uncertainty experienced by staff where policies and avenues for deliberation are absent and clinical leaders abdicate responsibility<br>Ways intimacy and sexuality were understood and perceived<br>How the need is expressed<br>Need of intimate relationship with another person<br>Expressing attention/affection to another resident<br>Other<br>resident indicated that the notion of the facility as 'home' fell short for her, that privacy was tokenistic<br>registered nurse acknowledged there was symbolic rather than actual privacy for residents |
| Note | |

| | |
|---|---|
| Title | Supporting residents' expression of sexuality: the initial construction of a sexuality assessment tool for residential aged care facilities |
| Author (Year) | Michael Bauer, Deirdre Fetherstonhaugh, Laura Tarzia, Rhonda Nay and Elizabeth Beattie (2014) |
| Aim | To describe the development process of a self-report tool for residential aged care facilities: SexAT. The tool aims to assist facilities to support the expression of residents' sexuality by: identifying areas where improvements in the environment, policies, procedures and practices, information provision and education/training may be required and; enabling monitoring of these areas over time |
| Met_Study Design | Mixed methods: interview+ focus group + delphi process. were conducted to explore participants' views and attitudes towards sexuality in aged care and the perceived needs and barriers to its expression |
| Met_Population | 46 staff members, 5 residents with and 11 without dementia, and 7 residents' family members |
| Met_Intervention | Sexuality assessment tool |
| Met_Primary_outcome | to help residential aged care facilities support the expression of sexuality of residents, both with and without dementia. |
| Results | Identifying sexuality needs:<br>• resident,<br>Discuss the effects of any medications on their ability to express their sexuality at any time |

| | |
|---|---|
| | Discuss how satisfied they are with the facility support for the expression of their sexuality and anything that may be impacting on it (continence, pain, lack of opportunity) with appropriately trained staff.<br>• family,<br>• formal caregiver -> facility<br>The SexAT (sexual assessment tool): Facility policies; Determining the needs of the Older Person; Staff Education and Training; Information and Support for Older People; Information and Support for Families; The Physical Environment; Safety and Risk Management.<br>Reasons why the need is not recognised<br>How the need is expressed<br>Other<br><br>The SexAT is designed to be self-administered by an aged care facility manager, or other senior staff member who knows the facility well and is familiar with the policies, procedures, and strategies currently in place.<br>Tool sections: Facility policies; Determining the needs of the Older Person; Staff Education and Training; Information and Support for Older People; Information and Support for Families; The Physical Environment; Safety and Risk Management.<br>Determining the needs of older person items:<br>• The facility uses a sexuality assessment tool for trained staff to identify residents' needs relating to sexual expression.<br>• Changes in sexual expression or behaviours that impinge on the rights of others (disinhibition, obscene gesturing, unwanted contact, abusive behaviour, stalking, sexual harassment etc.) are documented and potential reasons investigated.<br>• Residents are given the opportunity to discuss the effects of any medications on their ability to express their sexuality at any time.<br>• Residents are given the opportunity to discuss how satisfied they are with the facility support for the expression of their sexuality and anything that may be impacting on it (continence, pain, lack of opportunity) with appropriately trained staff.<br>• Residents are asked how satisfied they are with their personal presentation and styling, and are offered opportunities to discuss, change, or adapt their personal style.<br>• Residents are asked if they are satisfied with their opportunities to socialise.<br>• The facility's promotional or marketing materials indicate that residents' rights to sexual expression will be supported (including residents who identify as gay, lesbian, bisexual, transgender or intersex).<br>The SexAT is an easy to use tool that fills a gap in aged care service delivery. It provides a framework for aged care organisations to identify how well their environment and practices recognise and support the rights of older people to express their sexuality, including for people with dementia |
| Note | |

| | |
|---|---|
| Title | Negotiating the Lack of Intimacy in Assisted Living: Resident Desires, Barriers, and Strategies |
| Author (Year) | Alexis A. Bender, Elisabeth O. Burgess, and Christina Barmon (2017) |
| Aim | to understand the creation of the personal and social meanings associated with intimacy, as well as the various processes that are operating in this setting |
| Met_Study Design | qualitative study |
| Met_Population | Atlanta USA, Assisted Living (AL) 23 people: residents, family, staff, and administrators. Dementia and cognitive impairment residents were excluded |
| Met_Intervention | open-ended, semistructured individual interviews with 23 residents |
| Met_Primary_outcome | explore barriers and facilitators to resident sexual expression in AL |
| Results | Identifying sexuality needs:<br>• resident,<br>• family, |

| | |
|---|---|
| | • formal caregiver<br>Reasons why the need is not recognised<br>Institutional and individual-level barriers: limited privacy, social rules and norms including gossip and the perception that dating and intimacy are forbidden in AL, availability of/access to desiderable partners.<br>Absence of policies.<br>Social sanctions from administration and family<br>How the need is expressed<br>Desire for companionship<br>Subtle expressions of desire<br>Flirting and joking<br>Trying to sit with someone or walk them to their room.<br><br>Other<br>Lack of intimacy |
| Note | Pubmed. Sample not generalizable |

| | |
|---|---|
| Title | "If you do not find the world tasty and sexy, you are out of touch with the most important things in life": Resident and family member perspectives on sexual expression in continuing care |
| Author (Year) | Julia Brassolotto , Lisa Howard, Alessandro Manduca-Barone (2020) |
| Aim | To understand residents' and family members' perceptions of sexual expression in continuing care homes |
| Met_Study Design | Qualitative study |
| Met_Population | Canada, adults who either lived in or had a family member living in a continuing care home for at least six months and had experience related to sexual expression in continuing care |
| Met_Intervention | Interviews |
| Results | Identifying sexuality needs:<br>• resident,<br>Some residents indicated that their expressions of sexuality have changed over time.<br>This was the result of different stages in relationships, changes in their bodies, and other life transition<br>Participants were unanimous that residents should have sexual autonomy to engage in certain expressions privately and consensually<br>• family,<br>• formal caregiver<br>Reasons why the need is not recognised<br>Lack of sexual and romantic life privacy<br>Lack of related communication in continuing care homes.<br>Residents and family members felt that there were no clear expectations, policies, guidelines, opportunities for conversations, or information about what types of supports were available to them.<br>Because it is unclear what is appropriate or inappropriate, we heard that many care homes err on the side of suppressing resident sexual expression. Unfortunately, this can result in discouraging wanted expressions and insufficient preparation for or stigmatizing responses to unwanted sexual expression.<br>How the need is expressed<br>use sex toys nightly<br>a resident's coming out<br>Touch and emotional intimacy<br>physical touch included non-medical touch<br>desire to look and feel good<br>making decisions about what to wear, hair style, social activities to participate, |

| | |
|---|---|
| | and more.<br>masturbation or sex toys use<br><br>Other<br>several residents indicated that their sexual lives ended when their spouses died. |
| Note | |

| | |
|---|---|
| Title | Management of Sexual Expression in Long-Term Care: Ombudsmen's Perspectives |
| Author (Year) | Laci J. Cornelison and Gayle M. Doll |
| Aim | to initiate an in-depth investigation of the management of sexual expression in institutional care based on interviews with long-term care ombudsmen. |
| Met_Study Design | Qualitative study |
| Met_Population | 31 ombudsmen |
| Met_Intervention | Interview |
| Results | Identifying sexuality needs:<br>• resident,<br>• family,<br>• formal caregiver<br>Ombudsmen intervened in cases based on dilemma or conflict due to risk. Ombudsmen assist in preparing facility staff to educate residents about sexually transmitted disease and safer sex practices. Ombudsmen are frequently contacted to consult on romantic relationships or sexual expression in people with dementia<br>Reasons why the need is not recognised<br>Lack of knowledge and education in the facility staff, residents and residents' families, or support systems. Lack of privacy<br>How the need is expressed<br>Other<br>Intimate relationships and the expression of sexuality may lead to personal danger, a threat to another person's rights, or danger to others living in the facility. |
| Note | |

| | |
|---|---|
| Title | Sexuality in nursing homes. Practice and Policy. |
| Author (Year) | Gayle M. Doll (2013) |
| Aim | examines the scope of resident sexuality, staff reactions to sexual behavior, and the policies and guidelines used in 91 nursing homes to address residents' sexual activity |
| Met_Study Design | Observational study. |
| Met_Population | USA. Administrators and social workers in Kansas nursing homes |
| Met_Intervention | Online Survey |
| Results | Identifying sexuality needs:<br>• resident,<br>• family,<br>support the facility actions<br>• formal caregiver<br>helping residents by consulting a supervisor (68,9%) or following the facility policies<br>Reasons why the need is not recognised<br>Forms of expression were considered as inappropriate due to a cultural bias against older people sexuality.<br>Staff members are often upset by sexual expression when they perceive themselves to be the targets of attention and advances.<br>How the need is expressed<br>Sexual talk, sexual act, implied sexual act, false allegations or abuse, romantic relationships. |

| | |
|---|---|
| | Other<br>Many of these responses indicate that sexual behaviours are seen as non-normative and are treated as problems. |
| Note | Nurses play a critical role in addressing these issues. Their attitudes and actions regarding resident sexuality can foster policy changes, acceptance of, and dignity for residents' sexual rights. |

| | |
|---|---|
| Title | Barriers to sexual expression in residential aged care facilities (RACFs): comparison of staff and residents' views |
| Author (Year) | Feliciano Villar, Montserrat Celdr_an, Josep Fab_a & Rodrigo Serrat .2014 |
| Aim | To explore and compare the views of both staff and residents about barriers to sexual expression in residential aged care facilities |
| Met_Study Design | A qualitative descriptive study⊚ |
| Met_Population | 53 staff members currently working in RACFs and 47 residents living in the same RACFs were interviewed for this study; Participants belonged to five different RACFs.<br>Most of the staff member subsamples were women (only five men)<br>age from 22–63 years<br>The subsample of residents comprised 27 women and 20 men. To participate in this study, residents had to meet the following inclusion criteria: permanent residence in the RACF for at least 6 months, age at least 65; no diagnosis of mild cognitive impairment, dementia of any kind or any other mental disorder. The final subsample ranged in age from 71–96 years (mean = 843; SD 586) and their educa- tional level was generally low: |
| Met_Intervention | interviews |
| Met_Primary_outcome | to ascertain what kinds of barriers to sexual expression in RACFs are identified by staff and by residents, comparing the two points of view |
| Results | Most participants were able to identify some kind of barrier to residents' sexual expression in RACFs. Only 19 people, all belonging to the residents subsample, did not identify any kind of barrier. Seven categories (kind of barriers) emerged in the analysis of barriers: lack of privacy, residents' attitudes, do not talk, poor health, staff attitudes, family interference and few opportunities. The most frequently mentioned barrier was the lack of privacy in RACFs, which prevents sexual encounters among residents.<br><br>According to participants, there is a belief that sexuality is socially and morally inappropriate in older age and this hinders the free expression of sexual needs and interest, which become linked to shame, embarrassment or guilt.<br><br>A third barrier mentioned by several staff members and a small number of residents had to do with a lack of communication about sexuality. Sexuality is an issue, which is not normally discussed in RACFs, and this contributes to its invisibility. This situation seems to benefit both residents and staff members, as sexuality is an issue that is perceived as difficult to address.<br><br>The fourth category that appeared in the analysis refers to health problems and diseases that limit the expression of sex- uality, particularly more so by staff members than residents. Some diseases are related to dependency and a lack of autonomy, while others have to do with sexual disabilities.<br><br>Families can be a source of restrictions and they tend to curtail or prevent the expression of sexual needs by their relatives who live in a RACF; The role of the family and their |

| | |
|---|---|
| | decisions regarding residents' sexual behaviour seems to be particularly relevant when the individual concerned has dementia |
| Note | |

| | |
|---|---|
| Title | Expressing sexuality in nursing homes. The experience<br>of older women: A qualitative study |
| Author (Year) | Domingo Palacios-Ceña, Rosa María Martínez-Piedrola, Marta Pérez-de-Heredia, Elisabet Huertas-Hoyas, Pilar Carrasco-Garrido, Cesar Fernández-de-las-Peñas, 2016 |
| Aim | The aim of the present study was to describe the lived experi-<br>ence of sexuality among older Spanish women residing in nursing homes. |
| Met_Study Design | A qualitative study |
| Met_Population | 20 female residents that lived permanently in a nursing home, without cognitive decline and who spoke Spanish. The women were included with a mean age of 83.4 years. Six were married, 1 was single and 13 were widows. |
| Met_Intervention | A semi-structured interview. |
| Met_Primary_outcome | The outcome was the identification of themes that represented the<br>female residents' experiences of sexual life in the nursing home. |
| Results | Three themes representing the experience of sexual life by female<br>residents were identified from the results:<br>1 expressing sexuality All residents confirmed that they continued to feel desire, but that their time had passed. On the other hand, they felt that sex was overvalued, whereas other ways of expressing feelings and emotions were undervalued. Many residents acknowledged that sex was not the only way open to them.<br>"Sex begins with a glance…the first thing is a gaze, stroking, shaking hands every day, knowing that you are accompanied. Sometimes sex is not necessary, now is when, at last, I have a deeper knowledge of my sexuality" (Par16, 82 years old, room).<br>2 sexuality as a duty All the residents in this study affirmed that sexuality is a part of a wife's marital duty. They acknowledged that their duties as a wife consisted in loyalty and obedience toward their husband, home maintenance tasks and taking care of the children. Furthermore, they reported feeling their needs were always secondary to their husband's "Many times I didn't want to do it, but I repeated to myself that it was my duty and his right .. Sometimes I wanted some- thing new, like for him to masturbate me, but he didn't take any notice or he looked at me as if I were dirty" (Par11, 90 years old, room).<br><br>3 respecting vows All the widowed residents spoke of how, once their husband had passed away, they never resumed sexual relations<br><br>The findings reveal that the residents included in this study<br>limited the expression of their sexuality although they continued to<br>feel desire.<br>The majority perceived sexuality as the 'duty' of a good<br>wife, and once their partner had passed away they avoided having<br>sexual relations. |
| Note | the sample size of the study is too small<br>BIAS: two data collection stages were included (each with different residents)<br>which may have influenced the results obtained |

| | |
|---|---|
| Title | Love, intimacy and sexuality in residential dementia care: A spousal perspective |
| Author (Year) | Tineke SM Roelofs; Katrien G Luijkx; Petri JCM Embregts , 2017 |
| Aim | Explore the experiences and needs of spouses of nursing home residents with dementia, regarding friendship, love, intimacy, and sexuality |
| Met_Study Design | qualitative design |

| | |
|---|---|
| Met_Population | nine participants were recruited from specialized, intensive 24-hour nursing home care units for people with dementia, form three RCFs |
| Met_Intervention | semi-structured conversational style + open questions |
| Met_Primary_outcome | to contribute to both research and practice regarding the perspectives of spouses of residents with dementia on what is a mostly neglected aspect of their lives.<br><br>contributes to filling the knowledge gap on experiences of love, intimacy, and sexuality by including spouses of residents in order to provide a more complete image. |
| Results | All spouses described a very difficult period, just before the admission of their loved ones to the RCF; this admission caused conflicting feelings of relief, anger and sadness. These conflicting feelings additionally influenced the experience of love for the spouses. Spouses in the current study made sense of this change in different ways. Some tended to define their relationship as a friendship, with a decrease in or even closure of intimate and sexual lives together. Others took the opportunity to fulfill their new caring role including love and intimacy. Love, emotional and physical intimacy were experienced as important aspects of being together as in contrast needs for merely physical sexuality were not shared. Love and togetherness were expressed in different ways: through protection or taking care of the loved one, frequent visits to the RCF and by acts of intimacy, such as cuddling and holding hands.<br>The barriers to experiencing intimacy in the context of a RCF were shared. In addition to feelings such as a lack of privacy, an unnatural situation and a lack of homeliness, participants also pointed at practical barriers, such as a shared bathrooms and small bedroom sizes. |
| Note | communication with caregivers was considered an important aspect, regarding the improvement of the possibilities to experience intimacy and sexuality by the spouses. It is interesting to further focus on this role of caregivers in future research and its implications for clinical practice. |

| | |
|---|---|
| Title | The influence of organizational factors on the attitudes of residential care staff toward the sexuality of residents with dementia |
| Author (Year) | Tineke S. M. Roelofs, Katrien G. Luijkx, Marielle C. M. Cloin and Petri J. C. M. Embregts. 2019 |
| Aim | to examine in a broad per- spective the attitudes of care staff toward the sexuality of residents with dementia, by examining the possible in- fluence of organizational factors on care staff attitudes. |
| Met_Study Design | A observational study |
| Met_Population | 191 employees + the sample was mostly female (n=179, 95.7%). Mean age was 40.8 years (range 18–64), and they had an aver- age of 16 years of tenure within care (range 1–43). Most of the participants completed an average vocational edu- cation and worked as direct caregivers. |
| Met_Intervention | survey + questionnaires + team meetings + Ordinary least square (OLS) |
| Met_Primary_outcome | connecting research and practice to elderly care |
| Results | Descriptive analyses have demonstrated that differences in attitude toward resident sexuality were found between groups of employees based on levels of education. Employees with high levels of education reported more positive attitudes than other employees; Therapists reported more positive attitudes than direct caregivers and other employees. Finally, attitudes were significantly influenced by the presence or absence of policy. Employees, who reported that policy regarding resident sexuality was not present, were found to have a more positive attitude than employees who did report that policy was present in their RCF organization.<br><br>Knowledge has a positive effect on attitudes.<br>More knowledge of resident sexuality goes together with a positive attitude toward resident sexuality. |

| | |
|---|---|
| | Care staff with a 'high vocational level' are found to have more positive attitudes toward resident sexuality than the 'average vocational level', the reference category. |
| Note | |

| | |
|---|---|
| Title | Need or right: Sexual expression and intimacy in aged care |
| Author (Year) | Margaret R. Rowntree , Carole Zufferey, 2015⊚ |
| Aim | to garner existing positive stories, and then build upon them to imagine new possibilities, in this case about sexual expression and intimacy in residential aged care. |
| Met_Study Design | qualitative design |
| Met_Population | total sample comprised 42 participants: 19 staff members (18 women and one man) and 23 community members (15 women and eight men). They ranged in age from 24 to 69 years. The staff member sample included occupational therapists, nurses, carers and lifestyle workers at base grade and more senior levels, including program managers. |
| Met_Intervention | interviews and focus groups: |
| Met_Primary_outcome | This paper attends to the discourses in the data that underpin and inform understandings about sexual expression in residential aged care. |
| Results | It is widely acknowledged by the staff members who volunteered in the study that the topic of residents' sexual expression in aged care is not handled well; it is 'side-stepped,' 'skimmed over' or 'taboo'.<br>A number of staff members raise the importance of normalizing residents' sexual expression.<br>Other suggestions involved the identification of a designated staff member/professional for relationships counseling and the procuring of sex worker services for interested residents. Staff members consider more is required than providing information, and suggest the documentation of residents' sexual needs and the development of specific care plans.<br><br>A discourse about 'rights' emerged within the community member data<br><br>Community members in both focus groups and interviews were passionate about their 'rights', emphasizing that an ideal facility would be responsive to sexual expression, be respectful, support autonomy and choice and provide privacy. Also drawing on the 'hotel' idea, one couple suggest that aged care facilities could make rooms more 'ordinary' and imagine access to a 'hotel room' with rights to be 'controlled by the couple'. |
| Note | |

| | |
|---|---|
| Title | Sexuality and intimacy among care home residents |
| Author (Year) | Paul Simpson; Maria Horne; Laura JE Brown; Tommy Dickinson; Christine Brown Wilson. 2016 |
| Aim | Identifies barriers to addressing sexuality and intimacy needs, and outlines some simple strategies to raise awareness of them among older care home residents and staff, thereby facilitating a discussion to enable such needs to be met |
| Met_Study Design | Qualitative study |
| Met_Population | residents and non-resident spouses in two "research- ready" care homes in north-west England and care home staff<br><br>Two focus groups – one in each care home – were facilitated by a male and female researcher working together, with nine participants in one group and seven in the other (n = 16). The discussions lasted for just over an hour and comprised mainly female care assistants aged from their 20s to their 60s. Each group also contained qualified nurses and at least one member of staff with significant dementia expertise. |
| Met_Intervention | semi-structured interview + focus groups |
| Met_Primary_outcome | Why sex is considered taboo for older people |

| | |
|---|---|
| | Identifying care home residents' interest in sexual matters How to address the sexual needs of care home residents |
| Results | Older people's experiences of sexuality and intimacy are affected by differences of gender, class, ethnicity and biography, and the accounts of those to whom we spoke indicate that lived experience is complex. When talking about sexual activity in par- ticular, residents spoke in ways that:<br>» Denied the need for sex;<br>» Expressed nostalgia for something they considered to belong in the past;<br>» Hinted at openness to sex and intimacy as ongoing concerns. |
| Note | |

| | |
|---|---|
| Title | The nursing team's performance towards the sexuality of institutionalized elderly women |
| Author (Year) | Larissa Venturini, Margrid Beuter, Marinês Tambara Leite, Jamile Laís Bruinsma, Carolina Backes. 2018 |
| Aim | Analyzing how the nursing team performs in facing the sexuality in the daily lives of institutionalized elderly women |
| Met_Study Design | A qualitative and descriptive study |
| Met_Population | Eighteen (18) nursing professionals who met the following selection criteria participated in the study: being a member of the nursing team of the institution, having been employed for at least 3 months, and not being on leave during the data collection period.<br>The age of nursing professionals between 25 and 42 years.<br><br>6 nurses and 12 nursing technicians, and 16 of them were females |
| Met_Intervention | semi-structured interview with:<br><br>-Closed questions for sociodemographic and labor characterization: training, specialization in the area of gerontology, gender, age, length of service and any connection to other institutions.<br><br>-Open questions addressing the nursing team's performance were also used such as: manifested situations of sexuality identified in the Institution; performance of the professionals in these situations and encountered difficulties. |
| Results | Although sex- uality should be recognized as an aspect that accompanies admission into the institutional setting, health professionals do not always feel comfortable addressing this issue.<br>The participants reported difficulties that may be related to attempts performed by nursing professionals in recognizing and managing situations related to sexuality, especially those that are homo affective.<br>The expression of some aspects of sexuality is considered with intimate and reserved connotation; thus, the impossibility of offering privacy to the residents is elucidated as an obstacle. The institutional organization, the large contingent of elderly women, shared rooms with other elderly women; no doors in some rooms and the need for providing care that the professionals need to offer are presented as the main difficulties related to privacy, and thus for manifestation of sexuality.<br>Control can be observed as a strategy adopted by professionals in their actions. The homo affective closeness by intimate or affective contact is cut back by the professionals through strategies that prioritize the spatial separation of the elderly involved, justified by the well-being of the other elderly women and for the serenity of the Institution, since the physical structure of the Institution allows for relocation to other rooms or areas. |

| | |
|---|---|
| | In this context, nursing professionals assume positions of supervision, distraction and separation of the manifestations of sexuality among the institutionalized elderly women. Thus, sexual behaviour is discouraged as much as possible, or simply disregarded. |
| Note | |

| | |
|---|---|
| Title | Relational autonomy in action: Rethinking dementia and sexuality in care facilities |
| Author (Year) | Elizabeth Victor, Laura Guidry-Grimes. 2018 |
| Aim | to clarify how to approach capacity determinations as it relates to sexual activity, propose how to theorize about patient autonomy in this context, and suggest some considerations for finding an ethically responsible and practically feasible way to respect the sexual rights of this population. |
| Met_Study Design | Review |
| Met_Population | individuals with early to moderate dementia in states of daily dependency on professional caregivers. |
| Met_Primary_outcome | The article critically examines existing empirical research on sexuality among persons with dementia, caregivers' attitudes, and institutional roadblocks to enabling residents' sexuality. |
| Results | Caregivers ought to attend to the different ways that commu- nication of choice could be evidenced by residents with dementia. In the absence of explicit verbalization, their interests could be inferred through patterns of behavior and dispositions.<br>The policies that block access to sexual expression result in compounded harm over time, making sexual autonomy increasingly morally urgent the longer opportunities for expression are denied.<br><br>Dementia can cause disinhibition, which sometimes leads to public sexual acts or the emergence of previously suppressed sexual preferences, given these considerations, caregivers need to know how to identify and weigh potential harms |
| Note | |

| | |
|---|---|
| Title | Effectiveness of a Sexuality Workshop for Nurse Aides in Long-Term Care Facilities |
| Author (Year) | Man-Hua Yang, Shu-Ting Yang, Tze-Fang Wang and Li-Chun Chang. 2021 |
| Aim | to improve elderly residents' quality of sexual life by enhancing nurse aides' knowledge and attitudes toward elderly sexuality through sexuality workshops. |
| Met_Study Design | quasi- experimental study |
| Met_Population | 68 nurse aides and 100 residents<br>Nurses having >3 months of working experience as a nurse aide, being willing to participate in the study, and being capable of completing questionnaires by themselves or after explanations in Mandarin or Taiwanese. The exclusion criteria were as follows: individuals who planned to resign or could not complete all four sessions of the workshop during the duration of the study.<br><br>For residents, the inclusion criteria were as follows: having normal cognitive function; being capable of communi- cating in Mandarin or Taiwanese; being willing to participate in the study; being capable of completing questionnaires by themselves or making selections on the questionnaires after explanation in Mandarin or Taiwanese; and having lived in the facility for >3 consecutive months. The exclusion criteria were |

| | |
|---|---|
| | as follows: individuals who were hospitalized during the study, planned to be discharged during the study, or had dementia |
| Met_Intervention | sexuality workshops, a group discussion for two hours per week for a total of four weeks + questionnaires |
| Results | The results of this study indicate that attending sexuality workshops has a positive effect on the sexual attitudes of nurse aides.<br><br>during the sharing and discussion section of the workshop, the nurse aides expressed that they could understand the existing sexual needs of the residents and have a positive attitude toward elderly sexuality after their sexual knowledge was improved. Therefore, it can be concluded that the sexuality workshop in the study had a positive effect on nurse aides' sexual attitude and was recognized to be effective.<br>After the intervention, the residents' quality of sexual life in the experimental group significantly improved at posttest and four weeks postintervention, indicating that cor- rect sexual knowledge and positive attitudes toward elderly sexuality by nurse aides improved the residents' quality of sexual life.<br><br>It was shown that the nurse aides' sexual knowledge and attitudes in the experimental group were superior to those in the control group after four sessions of a sexuality work- shop intervention, and the effects persisted at four weeks postintervention |
| Note | |
